# Carbapenem-resistant *Acinetobacter baumannii* carrier detection: a simple and efficient protocol

**DOI:** 10.1128/spectrum.04062-23

**Published:** 2024-03-01

**Authors:** Jonathan Lellouche, Alona Keren-Paz, Reut Rov, Reut Efrati Epchtien, Sammy Frenk, Amichay Hameir, Elizabeth Temkin, David Schwartz, Yehuda Carmeli

**Affiliations:** 1 National Institute for Antibiotic Resistance and Infection Control, Ministry of Health, Tel Aviv, Israel; 2 Adelson School of Medicine, Ariel University, Ariel, Israel; Johns Hopkins University, Baltimore, Maryland, USA

**Keywords:** carbapenem-resistant *Acinetobacter baumannii*, screening, carrier detection

## Abstract

Timely detection of carbapenem-resistant *Acinetobacter baumannii* (CRAB) carriers is essential to direct infection control measures. In this work, we aimed to develop a practical protocol to detect CRAB from screening samples. To choose a selective medium that detects CRAB with high sensitivity and specificity, 111 *A*. *baumannii* clinical isolates were inoculated on three types of agar: mSuperCARBA (SC), CHROMagar Acinetobacter (CaA), and modified CHROMagar Acinetobacter (mCaA) containing 4.5 mg/mL meropenem. SC was non-selective, CaA was the most sensitive (100%), but only moderately specific (72%), and mCaA was highly specific (97%) and sensitive (98%). Confirmation of the carbapenem-resistant phenotype using PCR-based detection of *bla*_OXA-23_, *bla*_OXA-24_, and *bla*_OXA-58_ genes was specific but not sensitive, detecting only 58% of CRAB isolates. Identification of *A. baumannii* using either *gyrB* or *bla*_OXA-51_ PCR was excellent. Next, we used the same methodology in routine screening for CRAB carriage. mCaA had the best yield, with high sensitivity but moderate specificity to differentiate between CRAB and other carbapenem-resistant organisms. Skin sampling using sponges and 6 hour enrichment was highly sensitive (98%), while other body sites had poor sensitivity (27%– 41%). Shorter incubation had slightly lower yield, and longer incubation did not improve the detection. Performing PCR for *bla*_OXA-51_ and *gyrB* on colonies growing on modified mCaA differentiated between CRAB and other species with high accuracy (98% and 99%, respectively). Based on our results, we present a procedure for easy and reliable detection of CRAB carriage using skin sampling, short enrichment, selection on mCaA, and PCR-based identification.

**IMPORTANCE:**

Carbapenem-resistant *Acinetobacter baumannii* (CRAB) is a substantial cause of nosocomial infections, classified among the most significant multidrug-resistant pathogens by the World Health Organization and by the US Centers for Disease Control. Limiting the spread of CRAB is an important goal of infection control, but laboratory methods for identification of CRAB carriers are not standardized. In this work, we compared different selective agar plates, tested the efficiency of *A. baumannii* identification by PCR for species-specific genes, and used PCR-based detection of common resistance genes to confirm the carbapenem-resistant phenotype. During a prospective study, we also determined the optimal sample enrichment time. Based on our results, we propose a simple and efficient protocol for the detection of CRAB carriage using skin sampling, short enrichment, selection on appropriate agar plates, and PCR-based identification, resulting in a turn-around time of 24 hours.

## INTRODUCTION

Carbapenem-resistant *Acinetobacter baumannii* (CRAB) is a substantial cause of nosocomial infections, including ventilator-associated pneumonia and bloodstream infections, and may cause severe skin and soft tissue infections, urinary tract infections, and secondary meningitis (1). The multidrug-resistant (MDR) phenotype of CRAB makes successful treatment of such infections extremely challenging. Therefore, CRAB is classified among the most significant MDR pathogens by the World Health Organization (2) and by the US Centers for Disease Control (3).

Once CRAB is introduced into a medical facility, it is hard to eradicate. It is highly tolerant to desiccation and antiseptic products, allowing it to persist in the hospital environment (4, 5). Therefore, limiting the spread of CRAB is an important goal of infection control. The reservoirs and sources of CRAB transmission in a healthcare setting are colonized patients and surfaces and fomites which they contaminate. Therefore, a cornerstone of CRAB-directed infection control interventions is early and accurate identification of carriers, in order to isolate them and to enhance cleaning and disinfection of their immediate surroundings. Laboratory methods for identification of CRAB carriers are not standardized. Neither the US CDC nor the European CDC provide specific guidance on methods to detect CRAB carriers. Traditionally, respiratory tract, throat, rectal, or skin samples are cultured to detect carriage. However, studies have shown poor test sensitivity, even when sampling multiple body sites (6). More recent studies showed that sampling large areas of the skin by sponge and using CRAB-selective media following enrichment improve the sensitivity of CRAB screening tests (7–11). After growth on selective media, organism identification and susceptibility testing are required before reporting a sample as CRAB positive. These culture-dependent techniques are relatively inexpensive and simple, which makes them feasible for large-scale screening when required. However, these methods have a relatively long turn-around time (TAT). PCR-based methods using *gyrB* (12) and *bla*_OXA-51-like_ (13) allow identification of *A. baumannii*. However, PCR tests targeting carbapenemases such as multiplex class-D-oxacillinase-encoding genes (*bla*_OXA-23-like_, *bla*_OXA-40-like_, *bla*_OXA-51-like_, and *bla*_OXA-58-like_) are limited in their ability to discriminate between carbapenem-susceptible and resistant strains. Therefore, PCR for CRAB screening has not gained wide use. In this study, we aimed to establish a laboratory workflow for detecting CRAB carriers in a setting where a high volume of tests is performed and timely results are required. Specifically, we aimed to evaluate the effectiveness of (i) the use of selective chromogenic plates and (ii) follow-up PCR for species identification and carbapenem resistance determination and (iii) establish the optimal enrichment time for screening samples to improve the sensitivity of CRAB detection.

## MATERIALS AND METHODS

### Study design

**Fig 1:**
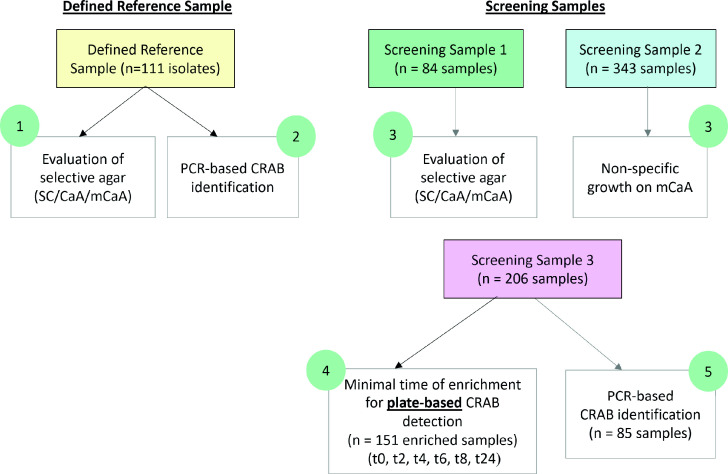
Study design. SC, mSuperCARBA; CaA, CHROMagar Acinetobacter; mCaA, modified CHROMagar *Acinetobacte*r; circles, aim number (see Materials and Methods for details).

The study components are shown in Fig. 1. Using a well-defined reference sample of 111 *A*. *baumannii* clinical isolates, we (Aim 1) compared three selective chromogenic plates for the growth of CRAB and carbapenem-susceptible *A. baumannii* and (Aim 2) tested the ability of PCR to correctly identify characterized *A. baumannii* isolates and to confirm carbapenem resistance. Next, we implemented the proposed methods for routine screening of CRAB carriers during three independent screenings in a post-acute care hospital (PACH) and (Aim 3) compared the detection of CRAB carriers using the chosen selective plates and (Aim 4) examined the optimal enrichment time required before inoculation on selective plates and (Aim 5) tested a PCR-based method to correctly identify *A. baumannii* and to confirm carbapenem resistance in screening samples. The results were used to develop an easy and efficient protocol for CRAB carrier detection.

### Samples (Figure 1)

#### Defined reference sample (Aims 1 and 2)

*A. baumannii* clinical isolates (*n* = 111) (Table S1) were collected between 2001 and 2017 and stored at the National Institute for Antibiotic Resistance and Infection Control in the Israeli Ministry of Health. Species identification was confirmed by mass spectroscopy (Microflex, Bruker, Bremen, Germany) before inclusion in this study. The meropenem (MEM) MIC for each isolate was verified by broth microdilution (BMD) in Muller-Hinton (MH), at concentrations ranging between 0.25 and 16 mg/L, according to CLSI guidelines (14). Results were interpreted according to CLSI breakpoints (15). The sample included 51 (46%) meropenem-susceptible (MEM MIC < 4 mg/L), 10 (9%) intermediate (MEM MIC = 4 mg/L), and 50 (45%) resistant (MEM MIC ≥ 8 mg/L) isolates. *Pseudomonas aeruginosa* ATCC 27853 (MIC ≤ 1 mg/L) and a carbapenem-resistant *P. aeruginosa* strain from our collection were used as controls.

**TABLE 1:** Selective agar comparison: growth of isolates by meropenem MIC (Aim 1)^*a*^

MIC MEM (mg/L)	Total isolates (*n*)	SC growth (*n*)	CaA growth (*n*)	mCaA growth (*n*)
≥0.25	3	33% (1)	0%	0%
0.5	3	100% (3)	0%	0%
1	20	100% (20)	15% (3)	0%
2	25	100% (25)	36% (9)	0%
4	10	100% (10)	50% (5)	20% (2)
8	12	100% (12)	100% (12)	92% (11)
16	36	100% (36)	100% (36)	100% (36)
32	2	100% (2)	100% (2)	100% (2)
Total	111	109	67	51

^*a*^ MIC was determined by BMD. CaA, CHROMagar Acinetobacter; mCaA, modified CHROMagar *Acinetobacter*; SC, mSuperCARBA plates.

All isolates were cryopreserved at −80°C and sub-cultured twice on blood agar (TSA + 5% sheep blood; Hy Laboratories, Rehovot, Israel) before investigations.

### Screening samples

On three separate occasions, screenings were conducted as part of an ongoing infection control intervention following CRAB outbreaks in a PACH and an acute care hospital. Skin and environmental samples were collected by sterile pre-moistened sponge (9) and enriched in 30 mL Brain Heart Infusion (BHI; Hy Laboratories, Rehovot, Israel) overnight at 35°C ± 2°C without agitation before inoculation. Rectal and buccal mucosa samples were collected using nylon swabs and enriched in 5 mL BHI overnight at 35°C ± 2°C without agitation before inoculation. Sputum samples were inoculated without enrichment.

For clinical purposes, a “carrier” was defined as a patient with one positive sample from any collection site. For the development of the protocol, a positive sample was defined as a sample with a positive result at any test condition (on any agar plate/at any enrichment time).

#### Sample 1: plate comparison (Aim 3)

Screening samples (*n* = 84) from 28 patients in a single PACH in the central district of Israel were collected. Skin, rectal, and sputum samples were obtained from each patient as previously described (8).

#### Sample 2: non-specific growth on modified CHROMagar *Acinetobacter* (Aim 3)

Screening samples (*n* = 343) were collected in the same PACH as above (*n* = 227) and in a single acute care hospital in northern Israel (*n* = 116). Environmental samples (*n* = 54) and samples from 98 patients (93 samples from the skin, 96 from the rectum, 21 from buccal mucosa of non-intubated patients, and 79 from sputum of intubated patients) were collected.

#### Sample 3: optimal body site, time for enrichment, and PCR-based methods (Aims 4 and 5)

Screening samples (*n* = 206) were collected from 55 PACH patients (55 from skin, 55 from the rectum, 41 from buccal mucosa, and 55 from sputum), in the same PACH as above.

All samples were transported to the laboratory at 4°C before processing. Samples were inoculated on selective agar plates (as described below) and incubated overnight at 35°C ± 2°C under aerobic conditions. Identification and susceptibility were confirmed by VITEK2 (bioMérieux), card N308, before a sample was considered positive.

### Selective media

We compared three selective chromogenic commercial plates: (i) mSuperCARBA (SC), which is selective for bacteria carrying most carbapenemases but not selective for CRAB (ii), CHROMagar Acinetobacter with a proprietary MDR supplement (CaA) (CHROMagar, Paris, France), which is selective for MDR *A. baumannii* and *A. baumannii* complex (Abc), and (iii) a modified version of the CHROMagar *Acinetobacter* (mCaA), supplemented with meropenem to a final concentration of 4.5 mg/mL to inhibit the growth of meropenem-sensitive and intermediate *A. baumannii* isolates. MH agar plates were used as a nonselective reference media.

### Agar plate evaluation (defined reference sample)

Cryopreserved samples were grown overnight on MacConkey agar plates (Hylabs, Israel). Two microliters of fresh inoculum containing 10^4^ CFU was inoculated on the test plates, with a spot diameter of 5–8 mm. Each isolate was tested in 4 replicates.

Following overnight incubation at 35°C ± 2°C, results were read. A positive result was recorded if colonies appeared in all replicates, and a negative result was recorded if no growth appeared in all replicates. In case of discrepancy between replicates, the test was repeated. *P. aeruginosa* ATCC 27853 (MEM MIC ≤ 1 mg/L) and *K. pneumoniae* G436 (MEM MIC = 16) strains were used as controls.

To calculate the sensitivity and specificity of the selective agar plates, we grouped intermediate and susceptible isolates together. Thus, the final sample consisted of 55% (61/111) non-resistant and 45% (50/111) resistant strains.

### Agar plate evaluation (screening sample 1)

Ten microliters of each sample was inoculated onto the three selective plates tested (SC, CaA, and mCaA) using a sterile loop. Skin and rectal samples were enriched before inoculation. Sputum samples were inoculated without enrichment. Identification and meropenem susceptibility of all suspect colonies that grew after overnight incubation were determined using VITEK2.

### PCR-based identification of *A. baumannii* and detection of carbapenemase genes

Genomic DNA was isolated and purified from several loop-fulls of colonies using a universal extraction system (STARMag 96 Universal Kit, Seegene, Seoul, Republic of Korea) on an automated Microlab NIMBUS workstation (Hamilton, Reno, NV).

Identification of *A. baumannii* to the species level was performed by testing for the presence of *bla*_OXA-51_ (F: TGTCTAAGGAAGTGAAGCGTG, R: AACTGTGCCTCTTGCTGAG) (16) and *gyrB* genes (F: GTTCCTGATCCGAAATTCTCG, R: AACGGAGCTTGTCAGGGTTA) as previously described (12). The *bla*_OXA-23_, *bla*_OXA-24_, and *bla*_OXA-58_ genes were detected by a multiplex reaction described previously by Woodford et al. (17). Three previously sequenced *A. baumannii* isolates from the National Institute for Antibiotic Resistance and Infection Control collection (F127, G324, and F79), harboring wild-type *bla*_OXA-51_ and *gyrB* genes, and either *bla*_OXA-23_ or *bla*_OXA-23_ + *bla*_OXA-24_, served as controls.

### Non-specific growth on modified CHROMagar *Acinetobacter* (screening sample 2)

All samples were inoculated onto mCaA plates as described above. PCR identification for *bla*_OXA-51_ and *gyrB* genes was performed for all red colonies that grew after overnight incubation at 35°C ± 2°C. Isolates with a negative PCR result were identified to the species level using VITEK2 (bioMérieux).

### Enrichment time experiments (screening sample 3)

Skin, rectal, and buccal mucosa samples (*n* = 151) that were enriched in broth as part of the screening protocol were sampled immediately upon inoculation and subsequently at 2, 4, 6, 8, and 24 hours. At each time point, 100 µL of BHI was spread on mCaA plates.

### Statistical methods

A test of proportions was used to compare the sensitivity of different anatomic sites for CRAB screening. One-way repeated measures analysis of variance was used to compare different enrichment time points.

## RESULTS

### Agar plate evaluation using a defined reference sample (Aim 1)

All isolates of the defined reference sample grew on the control MH agar plates without antimicrobial agents. Increasing the meropenem concentration in CHROMagar *Acinetobacter* plates eliminated the growth of meropenem-susceptible and reduced the growth of meropenem-intermediate *A. baumannii* (Table 1).

Compared with the susceptibility as determined by BMD, CHROMagar Acinetobacter was the most sensitive (100%) but only moderately specific (72%) (Table 2). Modified CHROMagar Acinetobacter was both highly specific (97%) and sensitive (98%). mSuperCARBA, which is intended to select for carbapenem-resistance *Enterobacterales* and not CRAB, was indeed non-selective, and all but two isolates grew on it.

**TABLE 2:** Selective agar comparison: selectivity and sensitivity (Aim 1)^*a*^

	SC	CaA	mCaA
Sensitivity	100%	100%	98%
(95% CI)	(0.9–1.0)	(0.9–1.0)	(0.9–1.0)
Specificity	3%	72%	97%
(95% CI)	(0–0.1)	(0.6–0.8)	(0.9–1.0)

^*a*^ CaA, CHROMagar Acinetobacter; CI, confidence interval; mCaA, modified CHROMagar Acinetobacter; SC, mSuperCARBA plates. Broth microdilution was used as the gold standard.

### PCR-based identification of CRAB using a defined reference sample (Aim 2)

Next, we assessed the ability of PCR-based methods to confirm the species identification of the isolates included in the defined reference sample. PCR analysis revealed that all isolates (*n* = 111) harbored both intrinsic *bla*_OXA-51_ and *gyrB* genes (Table 3). We then tested whether any of the three common OXA carbapenemase genes can serve as an indicator for the resistant phenotype. In 58.0% (29/50) of the resistant isolates, one or more carbapenemases were detected: *bla*_OXA-23_ (*n* = 13), *bla*_OXA-24_ (*n* = 10), and *bla*_OXA-23_+ *bla*_OXA-24_ (*n* = 6) (Table 3). *bla*_OXA-58_ was not detected in this sample. In 42% (21/50) of the resistant isolates, only *bla*_OXA-51_ was detected. No *bla*_OXA-23_ or *bla*_OXA-24_ was detected in any of the susceptible or intermediate isolates.

**TABLE 3:** PCR-based detection of *bla*-_OXA_ genes in a defined reference sample (Aim 2)^*a*^

		*bla* gene				
MEM MIC (mg/L)	No. of isolates, *n*	OXA-23, ***n*** (%)	OXA-24, *n* (%)	OXA-23 + 24, *n* (%)	OXA-58 *n* (%)	Any *bla* gene, *n* (%)
0.25	3	0	0	0	0	0
0.5	3	0	0	0	0	0
1	20	0	0	0	0	0
2	25	0	0	0	0	0
**Total susceptible**	**51**	**0**	**0**	**0**	**0**	**0**
4	10	0	0	0	0	0
**Total intermediate**	**10**	**0**	**0**	**0**	**0**	**0**
8	12	0	2 (16.0)	0	0	2 (16.7)
16	36	12 (33.3)	8 (22.2)	6 (16.7)	0	26 (72.2)
≥32	2	1 (50.0)	0	0	0	1 (50.0)
**Total resistant**	**50**	**13** (**26.0**)	**10** (**20.0**)	**6** (**12.0**)	**0**	**29** (**58.0**)
Total	111					

^*a*^ MIC (mg/L) was determined by BMD.

### Evaluation of selective plates during routine screening for CRAB carriage (Aim 3)

Screening sample 1 identified 57% (16/28) of the patients as carriers, based on at least one positive sample out of the three taken. CRAB was detected in 31/84 (37%) samples (Table 4). Based on visual inspection of growth on the plate, mCaA detected all positive samples and CaA detected 30/31 (97%) positive samples. SC detected 20/31 (65%) positive samples. Of the remaining 11 samples, 5 showed mixed growth and 6 showed no growth on SC. In seven CRAB-negative samples, non-CRAB growth was detected on SC: one meropenem-susceptible *A. baumannii* isolate, four KPC-producing *K. pneumoniae* isolates, one *Stenotrophomonas maltophilia* isolate, and one case of mixed growth of several *Enterobacterales* sp.

**TABLE 4:** Selective agar evaluation of CRAB screening samples (Aim 3)^*a*^

	Positive (*n*)	% of TP	Negative (*n*)	% of TN^*b*^	Mixed growth	CPE	Sensitive Ab
SC	20	65%	52	87%	6	5	1
CaA	30	97%	54	100%	0	0	0
mCaA	31	100%	53	100%	0	0	0

^*a*^ A total of 84 screening samples were tested. Ab, *A. baumannii*; CaA, CHROMagar *Acinetobacter*; CPE, carbapenem producing Enterobacterales; mCaA, modified CHROMagar *Acinetobacter*; SC, mSuperCARBA plates.

^*b*^ Refers to the percentage of all samples classified as negative that either did not grow at all or that were identified as non-*A. baumannii* after isolation, identification, and susceptibility by VITEK2. TN, true negative; TP, true positive.

In screening sample 2, 37% (36/98) of the patients was identified as carriers, based on at least one positive sample out of the three taken. Growth on modified CHROMagar Acinetobacter agar occurred in 135/343 samples. CRAB was detected in 72/135 (53%) of the positive samples and non-specific growth in 63/135 (47%) positive samples (Table 5). The majority of these non-CRAB isolates were species with intrinsic resistance to meropenem (e.g., *S. maltophilia* and *Elizabethkingia meningoseptica*). Thus, in a screening sample with CRAB positivity of ~35%, one would expect 50% of the isolates growing on mCaA plates to be CRAB.

**TABLE 5:** Non-specific growth on modified CHROMagar *Acinetobacter* plates (Aim 3)^*a*^

Species	Number of samples (%)	Reason for growth
*Stenotrophomonas maltophilia*	22 (6.4)	IR
*Elizabethkingia meningoseptica*	19 (5.5)	IR
*Pseudomonas aeruginosa*	7 (2.0)	MEM MIC ≥ 16 mg/L
*Pseudomonas putida*	4 (1.2)	MEM MIC ≥ 16 mg/L
*Chryseobacterium indologenes*	4 (1.2)	IR
*Klebsiella pneumoniae*	2 (0.6)	MEM MIC ≥ 16 mg/L
*Burkholderia cepacia complex*	1 (0.3)	MEM MIC ≥ 16 mg/L
*Chryseobacterium gleum*	1 (0.3)	IR
*Leclercia adecarboxylata*	1(0.3)	MEM MIC ≥ 16 mg/L
*Salmonella enterica*	1 (0.3)	UNK
*Shewanella putrefaciens*	1 (0.3)	UNK
Total	63 (18.4)	

^*a*^ Organisms other than CRAB identified following inconclusive initial visual inspection on modified CHROMagar *Acinetobacte*r plates based on colony color and suspected as CRAB. A total of 343 samples—screening (*n* = 289) and environmental (*n* = 54)—were evaluated. IR, intrinsic resistance; UNK, reason for growth unknown.

### Comparison of optimal sampling site for routine screening for CRAB carriage

Out of the 55 patients in screening sample 3, 44 (80%) were CRAB positive in at least one body site, yielding 91/206 positive samples. The yield differed by body site, with 98% (43/44) of CRAB-positive patients detected by skin samples. Sputum, rectal, and buccal mucosa samples were less sensitive (29%, 27%, and 41%, respectively, *P* < 0.001 for each site compared to skin).

### Evaluation of optimal time for sample enrichment before inoculation (Aim 4)

**Fig 2:**
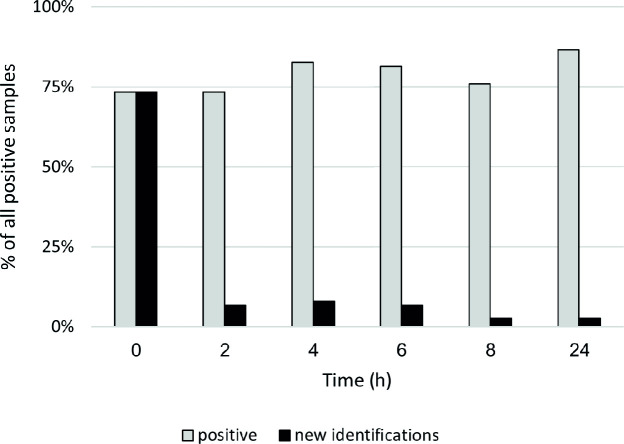
Association between enrichment time and CRAB detection (Aim 4). Gray, all positive samples detected at the specified time point; black, positive samples detected for the first time at the specified time point**.**

We defined “positive” as a sample yielding a positive result at one or more time points tested. Among the 151 samples tested repeatedly while undergoing enrichment, 75 were positive for CRAB at least at one time point. There was no single time point at which all positive samples were detected. As shown in Fig. 2, detection increased over time; however, when comparing rates of detection at single time points, the differences were not statistically significant: *P* = 0.096 for 0 h vs 24 h, *P* = 0.405 for 0 h vs 6 h, and *P* = 0.864 for 6 h vs 24 h.

### PCR-based identification of CRAB for routine screening for CRAB carriage (Aim 5)

To determine whether the PCR-based method could be used for species identification as part of a screening protocol, we performed PCR on 85 CRAB isolates. Most colonies tested positive for the presence of *bla*_OXA-51_ and *gyrB* (98% and 99%, respectfully), confirming them as *A. baumannii*. When tested for the presence of *bla*_OXA-23/24_, 58% (49/85) of the samples tested positive: 39% was positive for *bla*_OXA-23_ and 18% for *bla*_OXA-24_ and one sample was positive for both (Table 6).

**TABLE 6:** PCR-based detection of *gyrB* and *bla*-_OXA_ genes during carrier screening (Aim 5)^*a*^

				*bla* gene				
	*gyrB,* *n* (%)	OXA-51, *n* (%)		OXA-23, *n* (%)	OXA-24, *n* (%)	OXA-23 + 24, *n* (%)	OXA-58, *n* (%)	Any resistance gene, *n* (%)
Positive	84 (99)	83 (98)		33 (39)	15 (18)	1 (1)	0	49 (58)

^*a*^ A total of 85 CRAB isolates were tested.

## DISCUSSION

**Fig 3:**
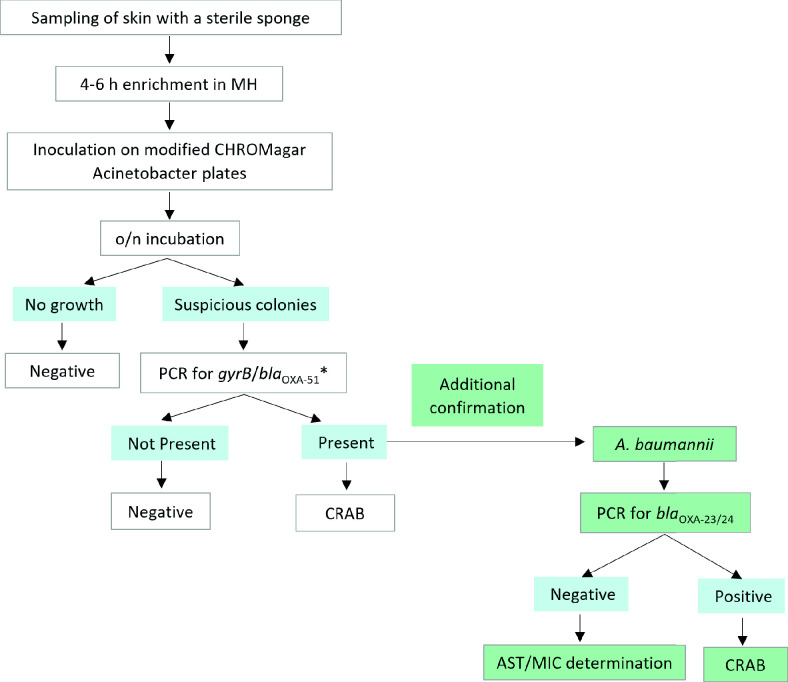
Proposed protocol. *This step can be substituted by identification using MALDI-TOF MS.

Failure to promptly isolate CRAB carriers or erroneously cohorting a CRAB-negative patient in a CRAB-positive environment can lead to transmission and outbreaks. Therefore, CRAB detection methods need to be fast and accurate. Our objective in this study was to assess different aspects of CRAB screening methods and to use the results to develop a CRAB screening protocol, which we present in Fig. 3. The proposed protocol, with reduced enrichment time and without the requirement for additional AST testing, results in a TAT of 24 hours: the first day for sampling, short enrichment, and inoculation on selective plates and the second day for identification and reporting the results.

The protocol is based on the following findings:

### Sample site and method

Consistent with previous reports (8), our results strongly suggest that the best site for sampling is skin, using a sterile pre-moistened sponge.

### Plates (Aims 1 and 3)

As reported previously (18) and validated in this study, CHROMagar Acinetobacter plates, but not SC, have good sensitivity and specificity for CRAB versus carbapenem-non-resistant *A. baumannii*. Increasing meropenem concentration in this media further increases specificity, without compromising sensitivity. Therefore, the use of appropriate plates can serve as an alternative to AST. Nevertheless, in our real-world study in a setting in which carbapenem resistance is endemic, almost 50% of the positive plates were the result of non-specific growth of other carbapenem-resistant species, highlighting the need for species identification.

### Enrichment time (Aim 4)

At no time point were we able to detect all CRAB-positive samples, and only by combining the results of all time points did we reach 100% CRAB detection. Interestingly, some samples were only positive early on and turned negative after prolonged enrichment. This might be due to the low initial frequency of CRAB and competition from other microbiota present in the sample. Thus, when comparing single time points, the enrichment protocol only slightly increased detection rates, from 73% without enrichment to 83% after 4 hours and finally to 87% after overnight enrichment, and none of the differences were statistically significant. When deciding on the optimal enrichment time, individual laboratories should weigh the trade-off between a shorter TAT (which accelerates isolation of carriers) and higher yield (which allows detection and isolation of more carriers).

### PCR-based detection (Aims 2 and 5)

According to our findings, *gyrB* and *bla*_OXA-51_ are specific and sensitive indicators for *A. baumannii* detection. As growth on modified CHROMAgar plates was highly specific, confirming identification of growing isolates by these PCR tests allows early reporting of CRAB carriage with high certainty. Although not tested in our study, MALDI-TOF MS rather than PCR can also be used for rapid identification of growing colonies (19, 20).

We found that the presence of antibiotic resistance genes *bla*_OXA-23_ and *bla*_OXA-24_ is a clear indicator of a meropenem-resistant phenotype. However, the absence of these genes did not serve as a reliable predictor of meropenem susceptibility in our sample. It is likely that additional mechanisms of resistance, for example, overexpression of the *bla*_OXA-51_ operon or the presence of emerging OXA variants not detected by the PCR reaction we employed, were common in our samples. We did not test all the isolates by whole genome sequencing to determine the resistance mechanism in those cases, and this is one limitation of our study. Therefore, detection of *bla*_OXA-23_ and *bla*_OXA-24_ may assist verification of carbapenem-resistant *A. baumannii*, but their absence is not sufficient to rule out CRAB, and thus, this method should not be used as the only tool for CRAB screening. Whenever possible, the PCR reaction aimed at identifying the mechanisms of resistance should be adjusted to reflect current and local epidemiology.

Compared with our previous work and the work of others (6, 8–10, 18), this study offers several novel results. First, the comparison between the previously described CaA medium and the new mCaA was not performed before; mCaA significantly outperformed CaA. Moreover, we show that mCaA reliably differentiated between CRAB and carbapenem-non-resistant *A. baumannii*, thus allowing reporting CRAB without additional susceptibility testing. Second, the optimal incubation time was not previously tested. Our results show that overnight incubation offers little advantage and that significantly shorted incubation time would be sufficient for most purposes.

In summary, skin sampling by sponge, short enrichment, and inoculation on modified CHROMagar *Acinetobacter* plates, followed by *gyrA* and/or *bla*_OXA-51_ PCR, is a highly sensitive and specific protocol for identification of CRAB carriers.

## SUPPLEMENTAL MATERIAL

Table S1 spectrum.04062-23-s0001.xlsx Defined reference sample isolates.

ASM does not own the copyrights to Supplemental Material that may be linked to, or accessed through, an article. The authors have granted ASM a non-exclusive, world-wide license to publish the Supplemental Material files. Please contact the corresponding author directly for reuse.

## References

[1] Antunes LCS, Visca P, Towner KJ. 2014. Acinetobacter baumannii: evolution of a global pathogen. Pathog Dis 71:292–301. doi:10.1111/2049-632X.12125 24376225

[2] Organization WH. 2017. WHO publishes list of bacteria for which new antibiotics are urgently needed. Saudi Med J.

[3] CDC. 2019. Antibiotic resistance threats in the United States, 2019 Center for disease control and prevention. Atlanta, Georgia

[4] Rajamohan G, Srinivasan VB, Gebreyes WA. 2009. Biocide-tolerant multidrug-resistant Acinetobacter baumannii clinical strains are associated with higher biofilm formation. J Hosp Infect 73:287–289. doi:10.1016/j.jhin.2009.07.015 19762119

[5] Jawad A, Seifert H, Snelling AM, Heritage J, Hawkey PM. 1998. Survival of Acinetobacter baumannii on dry surfaces: comparison of outbreak and sporadic isolates. J Clin Microbiol 36:1938–1941. doi:10.1128/JCM.36.7.1938-1941.1998 9650940 PMC104956

[6] Marchaim D, Navon-Venezia S, Schwartz D, Tarabeia J, Fefer I, Schwaber MJ, Carmeli Y. 2007. Surveillance cultures and duration of carriage of multidrug-resistant Acinetobacter baumannii. J Clin Microbiol 45:1551–1555. doi:10.1128/JCM.02424-06 17314222 PMC1865886

[7] Nutman A, Lerner A, Fallach N, Schwartz D, Carmeli Y. 2019. Likelihood of persistent carriage of carbapenem-resistant Acinetobacter baumannii on readmission in previously identified carriers. Infect Control Hosp Epidemiol 40:1188–1190. doi:10.1017/ice.2019.210 31379306

[8] Nutman A, Temkin E, Lellouche J, Ben David D, Schwartz D, Carmeli Y. 2020. Detecting carbapenem-resistant Acinetobacter baumannii (CRAB) carriage: which body site should be cultured? Infect Control Hosp Epidemiol 41:965–967. doi:10.1017/ice.2020.197 32618523 PMC7511923

[9] Nutman A, Lerner A, Schwartz D, Carmeli Y. 2016. Evaluation of carriage and environmental contamination by carbapenem-resistant Acinetobacter baumannii. Clin Microbiol Infect 22:949. doi:10.1016/j.cmi.2016.08.020 27596532

[10] Moran-Gilad J, Schwartz D, Navon-Venezia S, Carmeli Y. 2012. Laboratory evaluation of the Eswab transport system for the recovery of carbapenem-resistant Acinetobacter baumannii. Eur J Clin Microbiol Infect Dis 31:1429–1433. doi:10.1007/s10096-011-1460-2 22068274

[11] Doi Y, Murray GL, Peleg AY. 2015. Acinetobacter baumannii: evolution of antimicrobial resistance-treatment options. Semin Respir Crit Care Med 36:85–98. doi:10.1055/s-0034-1398388 25643273 PMC4465586

[12] Higgins PG, Wisplinghoff H, Krut O, Seifert H. 2007. A PCR-based method to differentiate between Acinetobacter baumannii and Acinetobacter genomic species 13TU. Clin Microbiol Infect 13:1199–1201. doi:10.1111/j.1469-0691.2007.01819.x 17850345

[13] Turton JF, Woodford N, Glover J, Yarde S, Kaufmann ME, Pitt TL. 2006. Identification of Acinetobacter baumannii by detection of the bla OXA-51-like carbapenemase gene intrinsic to this species. J Clin Microbiol 44:2974–2976. doi:10.1128/JCM.01021-06 16891520 PMC1594603

[14] CLSI. 2018. M07 methods for dilution antimicrobial susceptibility tests for bacteria that grow Aerobically. 11th Edition. Clinical and Laboratory Standards Institute.

[15] CLSI. 2021. M100 performance standards for antimicrobial susceptibility testing. Clinical Laboratory Standard Institute. 10.1128/JCM.00213-21 PMC8601225 34550809

[16] Huang XZ, Cash DM, Chahine MA, Nikolich MP, Craft DW. 2012. Development and validation of a multiplex TaqMan real-time PCR for rapid detection of genes encoding four types of class D carbapenemase in Acinetobacter baumannii. J Med Microbiol 61:1532–1537. doi:10.1099/jmm.0.045823-0 22878252

[17] Woodford N, Ellington MJ, Coelho JM, Turton JF, Ward ME, Brown S, Amyes SGB, Livermore DM. 2006. Multiplex PCR for genes encoding prevalent OXA carbapenemases in Acinetobacter spp. Int J Antimicrob Agents 27:351–353. doi:10.1016/j.ijantimicag.2006.01.004 16564159

[18] Moran-Gilad J, Adler A, Schwartz D, Navon-Venezia S, Carmeli Y. 2014. Laboratory evaluation of different agar media for isolation of carbapenem-resistant Acinetobacter spp. Eur J Clin Microbiol Infect Dis 33:1909–1913. doi:10.1007/s10096-014-2159-y 24865248

[19] Espinal P, Seifert H, Dijkshoorn L, Vila J, Roca I. 2012. Rapid and accurate identification of genomic species from the Acinetobacter baumannii (Ab) group by MALDI-TOF MS. Clin Microbiol Infect 18:1097–1103. doi:10.1111/j.1469-0691.2011.03696.x 22085042

[20] Marí-Almirall M, Cosgaya C, Higgins PG, Van Assche A, Telli M, Huys G, Lievens B, Seifert H, Dijkshoorn L, Roca I, Vila J. 2017. MALDI-TOF/MS identification of species from the Acinetobacter baumannii (AB) group revisited: inclusion of the novel A. seifertii and A. dijkshoorniae species. Clin Microbiol Infect 23:210. doi:10.1016/j.cmi.2016.11.020 27919649

